# Supplementary material for: Predictors and number of antenatal care visits among reproductive age women in Sub-Saharan Africa further analysis of recent demographic and health survey from 2017–2023: Zero-inflated negative binomial regression
Source: PLoS One. 2024 Oct 22;19(10):e0302297. doi: 10.1371/journal.pone.0302297 (PMC11495606; doi:10.1371/journal.pone.0302297)
Supplement: S1 File — (DOCX) [file pone.0302297.s001.docx]

Supplementary file 1: The country included in the analysis with the recent round of DHS from 2017-2023

|  | Country code | Survey data set | Year | Country name |
| --- | --- | --- | --- | --- |
| 1 | BG | Demographic Health survey | 2018 | Brundi |
| 2 | BF | Demographic Health survey | 2021 | Brukinafaso |
| 3 | CM | Demographic Health survey | 2022 | Cameron |
| 4 | CL | Demographic Health survey | 2021 | Cot divar |
| 5 | ET | Demographic Health survey | 2019 | Ethiopia |
| 6 | GA | Demographic Health survey | 2021 | Gabon |
| 7 | GM | Demographic Health survey | 2020 | Gambia |
| 8 | GN | Demographic Health survey | 2018 | Guina |
| 9 | KE | Demographic Health survey | 2022 | Kenea |
| 10 | LB | Demographic Health survey | 2020 | Liberia |
| 11 | MD | Demographic Health survey | 2021 | Madagascar |
| 12 | ML | Demographic Health survey | 2018 | Mali |
| 13 | MR | Demographic Health survey | 2021 | Maurtie |
| 14 | NG | Demographic Health survey | 2018 | Nigeria |
| 15 | RW | Demographic Health survey | 2020 | Rwanda |
| 16 | SN | Demographic Health survey | 2019 | Senegal |
| 17 | SL | Demographic Health survey | 2019 | Seriee Lion |
| 18 | TZ | Demographic Health survey | 2022 | Tanzania |
| 19 | ZM | Demographic Health survey | 2018 | Zambia |
