# Supplementary material for: Predictors and number of antenatal care visits among reproductive age women in Sub-Saharan Africa further analysis of recent demographic and health survey from 2017–2023: Zero-inflated negative binomial regression
Source: PLoS One. 2024 Oct 22;19(10):e0302297. doi: 10.1371/journal.pone.0302297 (PMC11495606; doi:10.1371/journal.pone.0302297)
Supplement: Attachment — Submitted filename: PLOS ONE REVIEWED MANUSCRIPT 2024.docx [file pone.0302297.s002.docx]

**Predictors and number of Antenatal Care Visit among Reproductive Age Women in Sub-Saharan Africa Further Analysis of Recent** **DHS from 2017 -2023: Zero-Inflated Negative Binomial Regression**

Abel Endawkie^1*^ Natnael Kebede^2^, Desale Behonegn Asmamw^3,^ Yawkal Tsega^4^,

**Affiliation**

^1^Department of Epidemiology and Biostatistics, School of Public Health, College of Medicine and Health Sciences, Wollo University, Dessie, Ethiopia.

^2^Department of Health Promotion, School of Public health College of Medicine and Health Sciences, Wollo University, Dessie, Ethiopia

^3^Department of Reproductive Health, Institute of Public Health, College of Medicine and Health Sciences, University of Gondar, Gondar, Ethiopia

^4^Department of Health System and Management, School of Public Health, College of Medicine and Health Sciences, Wollo University, Dessie, Ethiopia

Corresponding author*	Email Adress	[abelendawkie@gmail.com](mailto:abelendawkie@gmail.com)

Natnael Kebede :NK: [natnaelkebedete@gmail.com](mailto:natnaelkebedete@gmail.com))

Desale Behonegn Asmamw : DBA: [desalebihonegn1988@gmail.com](mailto:desalebihonegn1988@gmail.com)

Yawkal Tsega: YT: [yawkaltsega@gmail.com](mailto:yawkaltsega@gmail.com)

**Abstract:**

**Background:** Antenatal care (ANC) is an important component of maternal and child health care. To reduce perinatal mortality and improve women’s experience of care, the World Health Organization (WHO) recommends pregnant women should attend at least eight (ANC) visits. However, in Sub-Saharan Africa, the mean number of ANC visits among reproductive age women using Demographic and Health Surveys (DHS) data after the new WHO recommendation is so far limited. Therefore, the study aimed to determine the mean number of ANC, proportion of at least eight ANC visits and its predictors among reproductive age women in Sub-Saharan Africa using recent (DHS) data from 2017-2023.

**Method:** Community-based cross-sectional study using secondary data analysis was conducted among 188,880 weighted reproductive age women in Sub-Saharan Africa using a recent round of DHS data from 2017-2023. Zero-inflated negative binomial regression was conducted and statistical significance was declared at P-value <0.05 and AIRR with 95% CI was reported.

**Result:** The mean number of ANC visits among reproductive age women in Sub-Saharan Africa was 4.08, 95%CI [4.07, 4.09] and 7.3% of women had eight or more ANC visits during pregnancy. Age of women, maternal and husband educational status, types of pregnancy, birth order, household size, number of under-five children, and wealth index were statistically significant with the numbers of ANC visits among reproductive age women in Sub-Saharan.

**Conclusion:** The mean number of ANC visit among reproductive age women in Sub-Saharan Africa is so far lower than the new WHO recommendation of ANC visit for a positive pregnancy experience. This study also highlights that, the proportion of at least eight ANC visits is low and there are still disparities in the mean of ANC visits across different regions of SSA. Age of women, maternal and husband educational status, types of pregnancy, birth order, household size, number of under-five children, and wealth index were predictors of the number of ANC visits in Sub-Saharan Africa. Therefore, strengthening efforts should be made to promote frequent antenatal care (ANC) visits, and efforts to improve maternal and husband’s educational status in Sub-Saharan Africa.

**Key words:** Numbers of ANC visit, Predictors, reproductive age women, Sub-Saharan Africa, DHS data, Zero-Inflated Negative Binomial Regression

# Introduction

Antenatal care (ANC) is an important component of maternal and child health care, aimed at improving the health of pregnant women and their unborn babies (1). It is a sequence of clinical test and interventions that i~~ntention to make certain the well-being of each the mother and the fetus at some point of being pregnant~~ (2, 3). The ~~updated~~ World Health Organization (WHO) recommends ~~that pregnant women should attend as~~ a minimum eight ANC visits to reduce perinatal mortality and improve women’s experience of care (4). Antenatal care constitutes vital healthcare for the duration of being pregnant, delivered both at a health care facility and inside the home, gambling a pivotal position in maternal and child fitness(4, 5). Globally , approximately 295,000 girls died from preventable causes related to pregnancy and childbirth, an average of 810 deaths per day (6). Most of these deaths (94%) occurred in areas where resources were limited and many could have been prevented(6). Sub-Saharan Africa accounted for more than two-thirds (196,000) of all maternal deaths globally(6). The global maternal mortality rate (MMR), which refers to maternal deaths per 100,000 live births, is about 216, with a massive 95% in developing countries(7). Many pregnant women in Africa do not receive ANC services (5, 8, 9) and underutilization of ANC visit may result in adverse maternal and neonatal outcomes, including maternal morbidity and mortality, preterm delivery, low birth weight, and neonatal mortality(6, 7). According to a different study finding, the factors of ANC utilization were educational statuses of women(10-12), marital status of the woman(11, 13), husband's occupation(13, 14) and level of education(13, 15), media exposure(11, 16), household wealth index(13, 17), community women's literacy(18), interval between births(10), transportation issues(19), and distance to the health facility(20). Even though, there are study on prevalence and associated factors ANC visit among reproductive age women in different countries of Sub-Saharan Africa, there is paucity of evidence related to mean number ANC visit and its predictors among reproductive age women ~~from 2017-2023 DHS data~~ using count model. Moreover, most of the studies were conducted using binary model, information may lost while categorizing women’s different numbers of visits in the same group. ~~and~~ investigating using the count model help to improve information loss and will provide the average number of ANC visit and its predictors among reproductive age women. ~~in Sub-Saharan Africa using recent DHS data from 2017-2023.~~ This study will highlight the proportion of reproductive age women who were utilizing at least eight ANC visits and also establish evidence for policy evaluators on the ANC utilization in SSA countries based on the new WHO recommendation of ANC visit for a positive pregnancy experience. Therefore, the study aims to determine mean number of ANC visit, proportion of at least eight ANC and its predictors among reproductive age women in Sub-Saharan Africa using recent DHS data from 2017-2023. ~~after the new WHO recommendation of ANC visit for a positive pregnancy experience.~~

# Method

**Study design**

The research used a cross-sectional study design based on secondary data from the recent DHS in sub-Saharan Africa.

## Study settings and population

The study was done in Sub-Saharan Africa (SSA) which is located in Continent Africa ~~which has~~ a diverse population. It ~~is located in Sub-Saharan, or Non-Mediterranean Africa, and the area and regions of the continent of Africa had lie south of the Sahara. These include Central Africa, East Africa, Southern Africa, and West Africa.~~ ~~Sub-Saharan Africa is a region that~~ includes a large area with over 40 countries, about 1 billion people, and a rich and diverse culture. The study was conducted using the most recent nationally representative cross-sectional DHS data available from 2017-2023 after 2016 new WHO recommendation of ANC visit for a positive pregnancy experience(4).

## Source and study population

The source population was all reproductive-age women who gave birth five years before each respective survey in Sub-Saharan Africa, whereas those in the selected Enumeration Areas (EAs) were the study population.

Data source: We extracted the dependent and independent variables from the recent DHS data of the birth record (BR) data set, which contains the full birth history of all women interviewed. The data set also includes information on pregnancy and postnatal care, as well as immunization, health, and nutrition data for children born in the last 5 years (21, 22). The DHS was a household survey that was conducted every five years in low and middle-income countries. It is a crucial data source for maternal health care utilization issues in these countries, as it collects data on various reproductive health issues such as marriage, antenatal care visits, postnatal visits, fertility preferences, and contraception (21). The data collected from a DHS survey was organized in a hierarchical structure, with households within a cluster forming the top level. The next level consists of household members, followed by interviewed women and men as a subset of household members. The bottom levels of the hierarchy include pregnancies and children of each interviewed woman(21, 22).

Sample size and sampling method: The sample size was determined from the birth to recode file ‘BR file’ from the DHS dataset of Sub-Saharan African countries that had at least one survey conducted between 2017 and 2023. DHS uses a two-stage stratified cluster sampling technique. In the first stage, a sample of enumeration areas (EAs) was selected independently from each stratum with proportional allocation stratified by residence (urban and rural). In the second stage, households were taken from the selected EAs using a systematic sampling technique (22). The final sample size was 191,344 [188,880 final weighted reproductive age women were included].

Total Enumeration areas (Clusters) =1692

Primary Sampling Unit

1,096,430 Reproductive Age Women Were Interviewed and ~~complied~~ compiled from 18 SSA countries from 2017-2023 DHS data coded as “v013”

Eligible women during the survey

191,361 were pregnant and gave birth during the survey coded as “m14”

**191,344 [188,880 final weighted reproductive age women were sampled]**

Women with Unknown number of ANC visit (17)

Total Household = 1,096,430

Secondary Sampling Unit

## Figure 1 Sampling and exclusion procedures to identify the final sample size of the study

## Inclusion and exclusion criteria

Those who were pregnant and gave birth 5 year before survey and whose gestational age and status of ANC visit were known. ~~Moreover~~, the study included women whose gestational age was documented at delivery and who did not get ANC during their pregnancy. Nevertheless, the study did not include women documented as pregnancy termination and whose gestational age was uncertain or unrecorded at the time pregnancy.

Study variables: The dependent variable of this study was the mean number of antenatal care visit among pregnant woman from 0–9/10 months of pregnancy until the data collection period counted as (0, 1, 2, 3, 4, 5, 6, 7, 8, 9, 10…... 20). The independent variables included socio-demographic and economic-related factors like (individual maternal age, educational status, marital status, religion, partner educational status household level factors like; sex of the household head , age of the household head, household wealth index, community level factors like; residence , community level wealth index, community level educational status, region of SSA) and obstetric factors like birth interval, types of pregnancy, previous cesarean section were independent variable .

## Measurement and Operational definitions

**Count regression**: Count Regression model used for the non-negative integer which was counted data as 0, 1, 2, 3…(23) .

## Data Processing and Analysis

Data ~~were~~ used ~~to~~ extracted, cleaned, coded, and analyzed using STATA version 17 Statistical software. Sample weights were done before further analysis, and descriptive statistics were described using frequencies, percentages, median, and interquartile range, and presented using tables, figures, and narratives.

**Poisson regression model**

The Poisson regression model is a statistical technique used to model count data as a function of a set of independent variables. It is often used to model the relationship between a count response variable and one or more predictor variables. The model assumes that the response variable follows a Poisson distribution and that the logarithm of its expected value can be modeled as a linear combination of the predictor variables. The standard Poisson regression model assumes that the observations are independent over time and that the mean and variance of the dependent variable are equal(23). But in the situation of over dispersion, the Poisson regression's premise failed that since the variance exceeds the mean (variance = 6.55 and mean =4.08). To address the over dispersion, the negative binomial regression model with an unobserved particular effect (random term or error term) for the parameter was chosen. The over-dispersion parameter in the negative binomial (NB) specification was tested using a Likelihood Ratio (LR) test for the parameter α (p-value < 0.001) in contrast to the Poisson model specification(24).

**Zero-Inflated Negative Binomial Regression**

Negative binomial regression was fitted to determine the predictors of mean number of ANC visit among reproductive age women during pregnancy in Sub-Saharan Africa using recent round of DHS data from 2017-2023. Due to the hierarchical nature of DHS data necessitated the calculation of distinct measures of variation in order to detect any potential clustering effect: the intra-class correlation coefficient (ICC) was calculated using following formula(25) ICC = $\frac{\delta2}{\delta2+ \pi2/ 3}$ and the ICC was 3.2% which is less than 10% which favors the individual level analysis using negative binomial regression analysis . To detect zero-inflation the Vuong test was tested (<0.001) which indicates there is statistical difference between zero-inflated negative binomial regression and standard negative binomial regression model. However, Vuong test is not sufficient and traditional criteria to decide the model; Zero-inflated negative binomial regression and standard negative binomial regression model were compared using information criteria. Because zero-inflated negative binomial regression accounts the over-dispersion and zero-inflation, zero-inflated negative binomial regression model has good log likelihood ratio test and AIC/BIC than standard negative binomial regression model.

In order to detect multi-linearity, the variance inflation factor (VIF) was evaluated; the fitted model's mean VIF was 1.5. The best-fit model was ultimately determined by comparing the models using the information criteria, and the model with the lowest AIC/BIC was chosen.

Ethical Approval: No ethical approval was needed because we had used the demographic and health survey which identifies all data before making it public, and the used DHS data sets are openly accessible. There are no variables at the level of human subjects in the World Pop geographic data. An authorization letter was requesting to download DHS data set and then obtained from CSA after requesting at <https://dhsprogram.com/>. The dataset and all methods of this study were conducted according to the guidelines laid down in the Declaration of Helsinki and based on DHS research guidelines.

**Result**

**Socio-demographic characters with mean number of ANC visit among reproductive age women** **in SSA**

The study included a weighted sample of 188,880 women of reproductive age. The mean age and standard deviation of these women were 29 ±7 respectively. Among the total number of women, 69,840 (36.4%) who were not educated had a mean number of 3.39 antenatal care (ANC) visits with a 95% confidence interval (CI) of [3.37, 3.40]. On the other hand, 9,044 (4.9%) women of reproductive age with higher education had a mean number of 5.94 ANC visits with a 95% CI of [5.88, 5.99]. The mean number of ANC visits was lower among women who lived in the poorest household wealth index (3.29) than those in the richest household wealth index (5.08). Urban dwellers had a higher mean number of ANC visits (4.7) than rural dwellers (3.68). Among women of reproductive age in Central Africa, 18,275 (9.8%), West Africa, 78,407 (42.0%), East Africa, 39,761 (21.3%), and South Africa, 50,163 (26.9%) had a mean number of ANC visits of 3.79, 4.11, 4.00, and 3.98 respectively (Table 1).

**Mean numbers of ANC visit among reproductive age women in SSA.**

Among women of reproductive age in Sub-Saharan Africa, the average number of antenatal care (ANC) visits was 4.08 with a standard deviation of 2.56 with 95% confidence interval of [4.07, 4.09]. Out of 188,880 women who were pregnant before the survey, 13579 (7.28%) had eight or more ANC visits between 2017 and 2023 in Sub-Saharan Africa (Table 2). The mean number of ANC visits was lowest in Central Africa (3.79) and highest in West Africa (4.11).

**Predictors of the number of antenatal care visits among reproductive age women in Sub-Saharan Africa using zero-inflated negative binomial regression analysis**

Different count models were fitted for model selection, and the Zero-inflated negative binomial Regression was preferred over the standard negative binomial regression model due to its lower AIC value. The AIC value for the Zero-inflated negative binomial regression was 92,777, while that of the standard negative binomial regression model was 94,818. In this study, maternal age, women and husband educational status, types of pregnancy, birth order, household size, number of under-five children, and household wealth index were statistically significant at p-value <0.05.

The frequency of ANC visits were 1.04 times [AIRR 1.04, 95%CI (1.01, 1.17)], 1.06 times [AIRR 1.06, 95%CI (1.03, 1.09)], 1.09 time[AIRR 1.09, 95% CI (1.05, 1.13)], 1.11 times [AIRR 1.11, 95% CI (1.07, 1.15)], 1.15 times [AIRR 1.15, 95% CI (1.1, 1.2), and 1.15 times (AIRR 1.15, 95% CI (1.07, 1.24) for women aged 20-24, 24-29, 30-34, 35-39, 40-44, and 45-49 more likely as compared to women of 15–19 years of age respectively. The number of ANC visits were more for women who have primary [AIRR, 1.04, 95% CI (1.02, 1.055)], secondary [AIRR, 1.04, 95% CI (1.02, 1.06)], and higher education [AIRR 1.08, 95% CI (1.04, 1.19)] as compared to women who have no education respectively. Women with partner who have primary, secondary, and higher education had more frequency of ANC visits [AIRR, 1.05, 95% CI (1.04, 1.07)], [AIRR, 1.06, 95% CI (1.04, 1.08)] and [AIRR 1.08, 95% CI (1.05, 1.12)] as compared with women with partner who have no education respectively. The frequencies of ANC among women who have unwanted pregnancy were decreased by 9% as compared to wanted pregnancy. The frequencies of ANC among women were decreased by 2.9% when the birth orders were increased [AIRR 0.971, 95% CI (0.97, 0.98)]. The frequencies of ANC among women were increased by 1% when the household size was increased [AIRR 1.01, 95% CI (1.00, 1.011)]. The frequencies of ANC among women were decreased by 2.7% when the number of under-five children was increased [AIRR 0.973, 95% CI (0.97, 0.98)]. The frequency of ANC visits were more among women from poorer [AIRR, 1.07, 95% CI (1.05, 1.1)], middle [AIRR, 1.09, 95% CI (1.07, 1.11)], richer [AIRR, 1.14, 95% CI (1.12, 1.17)], and richest [AIRR, 1.27, 95% CI (1.23, 1.30)] as compared with women from poorest household wealth index respectively (Table3).

Discussion

This research was conducted a statistical analysis using zero-inflated negative binomial regression to determine the average number of antenatal care (ANC) and its predictors among 188,880 reproductive age women in Sub-Saharan Africa using recent rounds DHS data from 2017-2023. Out of 188,880 women of reproductive age who were pregnant before the survey, 7.28% ~~of~~ had eight or more antenatal care visits during pregnancy ~~in Sub-Saharan Africa from 2017 to 2023~~ which is slightly higher than previous study finding (26). ~~and also~~ the mean numbers of ANC visit among reproductive age women in Sub-Saharan Africa was 4.08, 95%CI [4.07, 4.09] which was slightly higher than the previous study finding in Ethiopia~~(3.28)~~ (27), and in SSA (3.83) using DHS data from 2008-2019(3.83)(28). This indicates a minor improvement in the frequency of ANC visits. However, it is below the WHO's updated recommendation for an ANC visit for a positive pregnancy experience in SSA(4). This indicates that most reproductive age women in Sub-Saharan Africa countries were not getting minimum ANC visit during pregnancy based on the new WHO’s guideline ~~of~~ ANC visit for a positive pregnancy experience(4). The WHO recommends that a woman should get at least eight ANC visits during pregnancy for a positive pregnancy experience. This study also found that there are still disparities in the mean number of ANC visits across different regions of SSA. These disparities could be due to inequalities in the accessibility of maternal health services, poor or absent transportation, inequality in the number of health care providers, and disparities in access to education. Additionally, the differences in the implementation of maternal and child health programs, women’s education, and the role of women in household wealth status across different regions of SSA could also contribute to these disparities.

In this study, maternal age , women and husband educational status, types of pregnancy, birth order, household size, number of under-five children, and household wealth index, were predictors of numbers of ANC visit which is similar with ~~the previous study finding (~~~~11-14~~~~,~~ ~~29-31~~~~).~~ The frequency of ANC visits were 1.04, 1.06 , 1.09, 1.11, 1.15 and 1.15 times more likely for women aged 20-24, 24-29, 30-34, 35-39, 40-44, and 45-49 as compared to women of 15–19 age respectively. The higher age of pregnant women was positively associated with numbers of ANC visit. This indicates that older pregnant women are more likely to schedule to get more numbers of ANC visits. This is similar ~~finding with other study conducted previously (~~~~27~~~~,~~ ~~28~~~~).~~ This may be due to as women age increase, they may experience birth-related complications and poor health conditions, which could lead them to demand more visits. Additionally, older women may have more experience with pregnancy care compared to younger women (15-19 years old). Correspondingly, educational status of pregnant women and her ~~husband had positively associated~~ with frequency of ANC visit. This was supported by other previous study finding (9, 12, 18, 28, 29). This may due to educated husbands may have better ability to convince their spouses to have more prenatal care during pregnancy because they have favorable knowledge about maternal and child health. One explanation would be that those women who have educated husbands will also likely to understand themselves ~~and possibly this may~~ due to support by their partner(husband) which may increase discussion to get maternal health service utilization (32). To promote frequent antenatal care (ANC) visits, efforts should be made to improve maternal and husband’s educational status, ~~intern as higher education levels encourage their support for frequent ANC visits and educated husbands may have better ability to convince their partners for frequent ANC visits in Sub-Saharan Africa.~~

~~The~~ more ~~frequency of ANC visit directly~~ associated with higher number of household member. The frequencies of ANC among women were increased by 1% when the household size was increased. The study did not, however, offer any, correlation that exists between the number of homes member and the number of ANC visit. a study conducted in Ethiopia ~~showed~~ supported this evidence(29). The frequencies of ANC among women were decreased by 2.9% when the birth orders were increased. This is supported by the previous research (28, 33). This may be due to increased confidence from prior birth experiences. When the number of under-five children increased, the frequency of ANC among women decreased by 2.7%. This is consistent with other study finding(34). This could be due to mothers becoming overwhelmed with caring for their children at home, which may lead to reduced ANC utilization. Number of ANC visit directly associated with higher levels of household wealth status as compared to women from poorest household wealth index (9, 12, 35). The possible reason may due to richest households wealth index will produce women who are more independent, educated, and confident when seeking maternal health services. The possible reason may due to ~~richest and educated community produce~~ women who are more independent, educated, and confident when seeking maternal health services. The number of ANC visit was higher among pregnant women who ~~have wanted pregnancy as compared to women who have unwanted pregnancy~~. This finding was similar with previous study finding (31, 36). This may be ~~explained~~ due to ~~mother who was ready for pregnancies~~ have better interest to care themselves and get maternal health service. . It is obvious that if the pregnancy was wanted women’s willingness to get health services would increase and there might be early detection of pregnancy, which in turn leads to early booking for ANC, and as a result, the frequency of ANC visits would increase.

The strength of this study is the use of national representative data, which allows it to be generalizable to all Sub-Saharan African countries. ~~pregnant women.~~ The DHS surveys collected through DHS surveys rely on self-reported information, which can be subject to recall bias or social desirability bias, ~~were~~ may be limitation of this study.

Conclusion

The mean ANC visit among reproductive age women in Sub-Saharan Africa is so far lower than the new WHO recommendation of ANC visit for a positive pregnancy experience. This study also highlights that proportion of at least eight ANC visits are low and there are still disparities in the mean number of ANC visits across different regions of SSA. In this study, age of women, maternal and husband educational status, types of pregnancy, birth order, household size, number of under-five children, and wealth index were predictors of number of ANC visit in Sub-Saharan Africa. Therefore, strengthening ~~efforts to promote frequent antenatal care (ANC) visits, and efforts to improve maternal and husband’s educational status, as higher education levels encourage their support for frequent ANC visits, and educated husbands may have better ability to convince their partners for frequent ANC visits in Sub-Saharan Africa.~~ We strongly recommend, the ~~governments of Sub-Saharan African countries should have economical support for women who are marginalized, including those who lack education, and poor to achieve positive pregnancy experience recommended by WHO.~~

**List of Abbreviation:** AIC: Akakian information Criteria, AIRR: Adjusted incidence rate ratio, ANC: Antenatal care, BIC: Bayesian information Criteria, CSA: central statistical agency, Demographic and Health Survey, EA: Enumeration Area, LR: log likelihood Ratio NBR: negative binomial regression, SSA: Sub-Saharan Africa, WHO: World Health Organization, ZINBR: Zero-inflated negative binomial regression

**Ethics approval and consent to participate:** No ethical approval was needed because we had used the demographic and health survey data which identifies all data before making it public and the used DHS data sets are openly accessible. There are no variables at the level of human subjects in the WorldPop geographic data. An authorization letter was requested to download DHS data set and then obtained from CSA after requesting at <https://dhsprogram.com>.

The dataset and all methods of this study were conducted according to the guidelines laid down in the Declaration of Helsinki and based on DHS research guidelines.

**Participant consent for publication**: No consent to publish was needed for this study as we did not use any details, images or videos related to individual participants. In addition, data used are available in the public domain.

**Data availability statement**: Data are available in a public, open access repository. Data for this study were sourced from Demographic and Health surveys (DHS) and available here: <https://dhsprogram.com/>

**Competing interests:** The authors declare that they have no competing interests.

- **Consent to publication:** Not applicable
- **Founding:** No funding source

**Author contributions**:

Data curation: AE Formal analysis: AE, Investigation: AE, Methodology: AE, Software: AE, Supervision: AE and YT, Validation and Visualization: AE, and YT, Writing – original draft: AE, Writing – review & editing: AE, NK, DB, and YT. The final manuscript has been read and approved by all writers. All authors accept full responsibility for the work and/or study's conduct, had access to the data, and made the decision to publish.

**Acknowledgments:** The authors are sincerely grateful to the Demographic Health Survey (DHS) program for providing us to use DHS dataset through their archives <https://dhsprogram.com/>

# Reference

1.	Organization. WH.	WHO recommendations on interventions to improve preterm birth outcomes. 2015.

2.	Organization WH. . WHO recommendations on maternal health: guidelines approved by the WHO Guidelines Review Committee. World Health Organization; . 2017.

3.	Kang Y, Chimanya K, Matji J, Garg A, Heidkamp R, Marshal Q, et al. Determinants of Minimum Dietary Diversity Among Children Aged 6-23 Months in 7 Countries in East and Southern Africa (P10-035-19). Current Developments in Nutrition. 2019 2019-06-01T00:00:00Z.

4.	WHO. New guidelines on antenatal care for a positive pregnancy experience. 2016.

5.	Jones A SB. The importance of early antenatal care. J Matern Health. . 2018;22(4):123-135.

6.	WHO. . Maternal mortality fact sheet. World Health Organization. 2022.

7.	Group. WB. Trends in Maternal Mortality: 2000 to 2017. [cited 2022 Jan 1]. . 2017.

8.	Kyei-Nimakoh M, Carolan-Olah M, McCann TV. Access barriers to obstetric care at health facilities in sub-Saharan Africa—a systematic review. Systematic Reviews. 2017 2017-06-06T00:00:00Z.

9.	Kisuule I, Kaye DK, Najjuka F, Ssematimba SK, Arinda A, Nakitende G, et al. Timing and reasons for coming late for the first antenatal care visit by pregnant women at Mulago hospital, Kampala Uganda. BMC Pregnancy and Childbirth. 2013 2013-05-25T00:00:00Z.

10.	Adhikari T, Sahu D, Nair S, Saha K, Sharma RK, Pandey A. Factors associated with utilization of antenatal care services among tribal women: A study of selected States. The Indian journal of medical research. 2016 2016-07-01T00:00:00Z.

11.	Belay A, Astatkie T, Abebaw S, Gebreamanule B, Enbeyle W. Prevalence and factors affecting the utilization of antenatal care in rural areas of Southwestern Ethiopia. BMC Pregnancy and Childbirth. 2022 2022-01-14T00:00:00Z.

12.	Halima SE-S, Eid SM, El-Bassioune WM. Prevalence and Determinants of First Antenatal Care Visit among Pregnant Women Attending Public Health Institutions at Damietta Governorate, Egypt. International Journal of Medical Arts (Print). 2021 2021-04-01T00:00:00Z.

13.	Adulo LA, Hassen SS. Magnitude and Factors Associated with Late Initiation of Antenatal Care Booking on First Visit Among Women in Rural Parts of Ethiopia. 2022 2022-06-27T00:00:00Z.

14.	Alamneh A, Asmamaw A, Woldemariam M, Yenew C, Atikilt G, Andualem M, et al. Trend change in delayed first antenatal care visit among reproductive-aged women in Ethiopia: multivariate decomposition analysis. Reproductive Health. 2022 2022-03-28T00:00:00Z.

15.	Fentaw KD, Fenta SM, Biresaw HB, Mulugeta SS. Time to first antenatal care visit among pregnant women in Ethiopia: secondary analysis of EDHS 2016; application of AFT shared frailty models. 2021 2021-11-09T00:00:00Z.

16.	Gebeyehu A, Muche A, Merid M. Trends of delayed first antenatal care visit among pregnant women in Ethiopia based on the EDHS 2000-2016 data: Multivariate decomposition analysis. 2020 2020-12-02T00:00:00Z.

17.	Dewau R, Muche A, Fentaw Z, Yalew M, Bitew G, Amsalu E, et al. Time to initiation of antenatal care and its predictors among pregnant women in Ethiopia: Cox-gamma shared frailty model. PLoS ONE. 2021 2021-02-05T00:00:00Z.

18.	Abebe GF, Alie M, Girma D, Mankelkl G, Berchedi AA, Negesse Y. Determinants of early initiation of first antenatal care visit in Ethiopia based on the 2019 Ethiopia mini-demographic and health survey: A multilevel analysis. PLoS ONE. 2023 2023-03-06T00:00:00Z.

19.	Tripathy A, Mishra PS. Inequality in time to first antenatal care visits and its predictors among pregnant women in India: an evidence from national family health survey. Scientific Reports. 2023 2023-03-22T00:00:00Z.

20.	Seid A, Ahmed M. Survival time to first antenatal care visit and its predictors among women in Ethiopia. PLoS ONE. 2021 2021-05-06T00:00:00Z.

21.	Statistics D. Guide-to-DHS-Statistics. Organization of DHS Data.

22.	Daniel J Corsi MN, Jocelyn E Finlay, SV Subramanian. Demographic and health surveys: a profil. International Journal of Epidemiology,. , December 2012, ; Volume 41, Issue 6Pages 1602–.

23.	Parodi S, Bottarelli E. Poisson regression model in epidemiology-an introduction [animal diseases]. Annali della Facoltà di Medicina Veterinaria-Università di Parma (Italy). 2006;26.

24.	Greene W. Functional forms for the negative binomial model for count data. Economics Letters. 2008;99(3):585-90.

25.	Austin PC, Merlo J. Intermediate and advanced topics in multilevel logistic regression analysis. Statistics in medicine. 2017;36(20):3257-77.

26.	Tessema ZT, Tesema GA, Yazachew L. Individual-level and community-level factors associated with eight or more antenatal care contacts in sub-Saharan Africa: evidence from 36 sub-Saharan African countries. BMJ open. 2022;12(3):e049379.

27.	Arefaynie M, Kefale B, Yalew M, Adane B, Dewau R, Damtie Y. Number of antenatal care utilization and associated factors among pregnant women in Ethiopia: zero-inflated Poisson regression of 2019 intermediate Ethiopian Demography Health Survey. Reproductive Health. 2022 2022-02-05T00:00:00Z.

28.	Gebeyehu FG, Geremew BM, Belew AK, Zemene M. Number of antenatal care visits and associated factors among reproductive age women in Sub-Saharan Africa using recent demographic and health survey data from 2008–2019: A multilevel negative binomial regression model. PLOS Global Public Health. 2022 2022-12-27T00:00:00Z.

29.	Kitaw TA, Haile RN, Kitaw TA, Haile RN. Time to first antenatal care booking and its determinants among pregnant women in Ethiopia: survival analysis of recent evidence from EDHS 2019. 2022 2022-12-09T00:00:00Z.

30.	Seyoum K, Tekalegn Y, Quisido B. Determinants and prevalence of early initiation of breastfeeding: Does the place of delivery matter? A comparative cross-sectional study based on the 2016 Ethiopian demographic and health survey data. Population Medicine. 2021 2021-12-23T00:00:00Z.

31.	Zegeye AM, Bitew BD, Koye D. Prevalence and determinants of early antenatal care visit among pregnant women attending antenatal care in Debre Berhan Health Institutions, Central Ethiopia. African journal of reproductive health. 2013 2013-12-01T00:00:00Z.

32.	Suandi D, Williams P, Bhattacharya S. Does involving male partners in antenatal care improve healthcare utilisation? Systematic review and meta-analysis of the published literature from low- and middle-income countries. International health. 2020 2020-09-01T00:00:00Z.

33.	Ochako R, Gichuhi W. Pregnancy wantedness, frequency and timing of antenatal care visit among women of childbearing age in Kenya. Reproductive health. 2016;13:1-8.

34.	Alibhai KM, Ziegler BR, Meddings L, Batung E, Luginaah I. Factors impacting antenatal care utilization: a systematic review of 37 fragile and conflict-affected situations. Conflict and Health. 2022;16(1):1-16.

35.	Jassey B. Prevalence and Determinants of Early Antenatal Care Visits Among Pregnant Women Attending Antenatal Care in Rural Gambia. JMMR (Jurnal Medicoeticolegal dan Manajemen Rumah Sakit). 2023 2023-04-15T00:00:00Z.

36.	Gebresilassie B, Belete T, Tilahun W, Berhane B, Gebresilassie S. Timing of first antenatal care attendance and associated factors among pregnant women in public health institutions of Axum town, Tigray, Ethiopia, 2017: a mixed design study. BMC Pregnancy and Childbirth. 2019 2019-09-18T00:00:00Z.

Table 1 Socio-demographic characters and pairwise comparisons of means of ANC visit with socio-demographic characters among reproductive age women in Sub-Saharan Africa using recent rounds of DHS from 2017-2023

| Variable | Frequency | Percentage | Mean# of ANC | Std. err. | 95%CI of Mean | |
| --- | --- | --- | --- | --- | --- | --- |
| Maternal age |  | | | | | |
| 15-19 | 13,775 | 7.4 | 3.74 | 0.02 | 3.70 | 3.78 |
| 20-24 | 41,107 | 22.0 | 3.99 | 0.01 | 3.96 | 4.01 |
| 25-29 | 47,359 | 25.4 | 4.07 | 0.01 | 4.04 | 4.09 |
| 30-34 | 38,107 | 20.4 | 4.12 | 0.01 | 4.09 | 4.15 |
| 35-39 | 28,678 | 15.4 | 4.10 | 0.01 | 4.08 | 4.13 |
| 40-44 | 13,317 | 7.1 | 3.93 | 0.02 | 3.88 | 3.97 |
| 45-49 | 4,263 | 2.3 | 3.62 | 0.04 | 3.55 | 3.69 |
| Maternal education |  | | | | | |
| No education | 69,840 | 37.4 | 3.39 | 0.01 | 3.37 | 3.40 |
| Primary education | 57,871 | 31.0 | 3.89 | 0.01 | 3.87 | 3.91 |
| Secondary education | 49,851 | 26.7 | 4.83 | 0.01 | 4.80 | 4.85 |
| Higher education | 9,044 | 4.9 | 5.94 | 0.03 | 5.88 | 5.99 |
| Maternal occupation status |  | | | | | |
| No have work | 67,612 | 37.0 | 3.80 | 0.01 | 3.78 | 3.82 |
| yes have work | 115,077 | 63.0 | 4.20 | 0.01 | 4.18 | 4.21 |
| Partner(husband ) education |  | | | | | |
| No education | 63,167 | 40.9 | 3.39 | 0.01 | 3.38 | 3.41 |
| Primary education | 40,584 | 26.3 | 3.85 | 0.01 | 3.83 | 3.88 |
| Secondary education | 38,121 | 24.7 | 4.83 | 0.01 | 4.81 | 4.86 |
| Higher education | 12,740 | 8.2 | 5.58 | 0.02 | 5.53 | 5.62 |
| Husband occupation status |  | | | | | |
| No have work | 15,954 | 10.2 | 3.60 | 0.02 | 3.56 | 3.64 |
| Yes have work | 140,460 | 89.8 | 4.07 | 0.01 | 4.06 | 4.09 |
| Wanted (pregnancy type) Pregnancy status |  |  |  |  |  |  |
| Wanted | 30,814 | 95.3 | 4.0 | 0.0 | 4.0 | 4.0 |
| Wnwanted Unwanted | 1,538 | 4.8 | 3.5 | 0.0 | 3.4 | 3.6 |
| fertility Preference |  | | | | | |
| Prefer | 120,982 | 67.9 | 4.06 | 0.01 | 4.05 | 4.08 |
| Not prefer | 57,128 | 32.1 | 4.00 | 0.01 | 3.98 | 4.02 |
| Media exposure |  | | | | | |
| Not Exposed | 94,346 | 51.6 | 3.66 | 0.01 | 3.65 | 3.68 |
| Exposed | 88,340 | 48.4 | 4.50 | 0.01 | 4.48 | 4.51 |
| Birth order |  | | | | | |
| 1 | 41,424 | 22.7 | 4.28 | 0.01 | 4.26 | 4.31 |
| 2 | 36,668 | 20.1 | 4.19 | 0.01 | 4.16 | 4.22 |
| 3 | 30,316 | 16.6 | 4.14 | 0.01 | 4.11 | 4.17 |
| 4 | 23,273 | 12.7 | 4.04 | 0.02 | 4.01 | 4.07 |
| 5 | 17,230 | 9.4 | 3.96 | 0.02 | 3.93 | 4.00 |
| 6 | 12,615 | 6.9 | 3.75 | 0.02 | 3.70 | 3.79 |
| 7 | 9,005 | 4.9 | 3.67 | 0.03 | 3.62 | 3.73 |
| 8 | 5,542 | 3.0 | 3.53 | 0.03 | 3.47 | 3.60 |
| 9 | 3,071 | 1.7 | 3.44 | 0.04 | 3.35 | 3.52 |
| 10+ | 3,545 | 1.9 | 3.06 | 0.04 | 2.98 | 3.14 |
| Sex of household head |  | | | | | |
| Male | 148,557 | 79.6 | 3.97 | 0.01 | 3.96 | 3.99 |
| Female | 38,048 | 20.4 | 4.20 | 0.01 | 4.18 | 4.23 |
| Age of household head |  | | | | | |
| 15-20 | 1,422 | 0.9 | 3.68 | 0.06 | 3.57 | 3.79 |
| 21-30 | 38,742 | 25.1 | 4.01 | 0.01 | 3.99 | 4.04 |
| 31-40 | 63,680 | 41.2 | 4.11 | 0.01 | 4.09 | 4.13 |
| 41-50 | 35,790 | 23.1 | 4.04 | 0.01 | 4.02 | 4.07 |
| 51-64 | 12,667 | 8.2 | 3.98 | 0.02 | 3.95 | 4.01 |
| >64 | 2,378 | 1.5 | 4.01 | 0.02 | 3.97 | 4.05 |
| Wealth index of household |  | | | | | |
| Poorest | 40,582 | 21.8 | 3.29 | 0.01 | 3.27 | 3.31 |
| Poorer | 38,814 | 20.8 | 3.71 | 0.01 | 3.69 | 3.74 |
| Middle | 37,252 | 20.0 | 4.05 | 0.01 | 4.03 | 4.08 |
| Richer | 36,709 | 19.7 | 4.44 | 0.01 | 4.42 | 4.47 |
| Richest | 33,249 | 17.8 | 5.11 | 0.01 | 5.08 | 5.14 |
| Residence |  | | | | | |
| Urban | 67,040 | 35.9 | 4.70 | 0.01 | 4.68 | 4.72 |
| Rural | 119,566 | 64.1 | 3.68 | 0.01 | 3.67 | 3.70 |
| Region of SSA |  | | | | | |
| Central Africa | 18,275 | 9.8 | 3.79 | 0.02 | 3.75 | 3.82 |
| West Africa | 78,407 | 42.0 | 4.11 | 0.01 | 4.09 | 4.12 |
| East Africa | 39,761 | 21.3 | 4.00 | 0.01 | 3.98 | 4.03 |
| South Africa | 50,163 | 26.9 | 3.98 | 0.01 | 3.96 | 4.00 |

Table 2 Number of reproductive age women experiencing antenatal care in SSA from 2017-2023

| #of ANC visit during pregnancy | Number of women who experienced ANC visit | Percent |
| --- | --- | --- |
| 0 | 17,172 | 9.2 |
| 1 | 7,460 | 4 |
| 2 | 14,755 | 7.91 |
| 3 | 35,241 | 18.89 |
| 4 | 44,744 | 23.98 |
| 5 | 26,478 | 14.19 |
| 6 | 18,824 | 10.09 |
| 7 | 8,351 | 4.48 |
| 8 | 5,983 | 3.21 |
| 9 | 2,553 | 1.37 |
| 10+ | 5,043 | 2.7 |
|  | Mean number of ANC visit | |
| Mean | 4.08, 95%CI( 4.07,4.09) |  |
| Standard deviation | 2.56 |  |
| Minimum | 0 |  |
| Maximum | 20 |  |

Table 3 Zero-inflated negative binomial regression analysis for predictors of the number of ANC visits in Sub-Saharan Africa using recent rounds of DHS data from 2017-2023 (n=188,880)

| Variable | Mean# of ANC | Model 1(NBR ) | | | Model 2(ZINBR) | | |
| --- | --- | --- | --- | --- | --- | --- | --- |
|  |  | AIRR | 95% CI of AIRR | | AIRR | 95% CI of AIRR | |
| Maternal age |  | | | | | | |
| 15-19 | 3.74 | Reference |  |  |  |  |  |
| 20-24 | 3.99 | 1.03 | 1.00 | 1.06 | 1.04 | 1.01 | 1.07** |
| 25-29 | 4.07 | 1.05 | 1.02 | 1.09 | 1.06 | 1.03 | 1.09*** |
| 30-34 | 4.12 | 1.08 | 1.04 | 1.11 | 1.09 | 1.05 | 1.13*** |
| 35-39 | 4.10 | 1.10 | 1.06 | 1.14 | 1.11 | 1.07 | 1.15*** |
| 40-44 | 3.93 | 1.14 | 1.09 | 1.19 | 1.15 | 1.10 | 1.20*** |
| 45-49 | 3.62 | 1.11 | 1.03 | 1.19 | 1.15 | 1.07 | 1.24*** |
| Maternal education |  | | | | | | |
| No education | 3.39 | Reference |  |  |  |  |  |
| Primary education | 3.89 | 1.015 | 0.9975 | 1.034 | 1.0364 | 1.0178 | 1.06*** |
| Secondary education | 4.83 | 1.047 | 1.0242 | 1.07 | 1.04 | 1.0175 | 1.063*** |
| Higher education | 5.94 | 1.092 | 1.0514 | 1.135 | 1.0774 | 1.037 | 1.12*** |
| Maternal occupation status |  | | | | | | |
| Have no work | 3.80 | Reference |  |  |  |  |  |
| Have work | 4.20 | 1.04 | 1.03 | 1.06 | 1.01 | 0.99 | 1.03 |
| Partner(husband ) education |  | | | | | | |
| No education | 3.39 | Reference |  |  |  |  |  |
| Primary education | 3.85 | 1.02 | 1.00 | 1.04 | 1.05 | 1.04 | 1.07*** |
| Secondary education | 4.83 | 1.04 | 1.02 | 1.06 | 1.06 | 1.04 | 1.08*** |
| Higher education | 5.58 | 1.08 | 1.05 | 1.12 | 1.08 | 1.05 | 1.12*** |
| Husband occupation status |  | | | | | | |
| No have work | 3.60 | Reference |  |  |  |  |  |
| Yes have work | 4.07 | 1.01 | 0.99 | 1.03 | 0.99 | 0.96 | 1.01 |
| Wanted pregnancy Pregnancy status |  | | | | | | |
| ~~w~~Wanted | 4.0 | Reference |  |  |  |  |  |
| Unwanted | 3.5 | 0.89 | 0.86 | 0.93 | 0.91 | 0.87 | 0.95*** |
| Fertility Preference |  | | | | | | |
| Prefer | 4.06 | Reference |  |  |  |  |  |
| Not prefer | 4.00 | 1.00 | 0.98 | 1.01 | 1.01 | 0.99 | 1.02 |
| Media exposure |  | | | | | | |
| Not Exposed | 3.66 | Reference |  |  |  |  |  |
| Exposed | 4.50 | 1.02 | 1.01 | 1.04 | 1.01 | 0.99 | 1.02 |
| Birth order |  | 0.97 | 0.97 | 0.98 | 0.971 | 0.97 | 0.98** |
| Household size |  | 1.01 | 1.00 | 1.01 | 1.01 | 1.00 | 1.01*** |
| Number of under-five children |  | 0.975 | 0.97 | 0.98 | 0.973 | 0.97 | 0.98*** |
| sex of household head |  | | | | | | |
| Male | 3.97 | Reference |  |  |  |  |  |
| Female | 4.20 | 1.00 | 0.98 | 1.02 | 1.00 | 0.98 | 1.02 |
| Age of household head |  | | | | | | |
| 15-20 | 3.68 | Reference |  |  |  |  |  |
| 21-30 | 4.01 | 1.00 | 0.92 | 1.08 | 1.00 | 0.92 | 1.08 |
| 31-40 | 4.11 | 1.00 | 0.93 | 1.09 | 1.00 | 0.92 | 1.09 |
| 41-50 | 4.04 | 1.01 | 0.93 | 1.10 | 1.01 | 0.93 | 1.09 |
| 51-64 | 3.98 | 0.95 | 0.88 | 1.03 | 0.96 | 0.88 | 1.04 |
| >64 | 4.01 | 0.99 | 0.91 | 1.07 | 0.99 | 0.91 | 1.08 |
| Wealth index of household |  | | | | | | |
| Poorest | 3.29 | Reference |  |  |  |  |  |
| Poorer | 3.71 | 1.08 | 1.06 | 1.10 | 1.07 | 1.05 | 1.10*** |
| Middle | 4.05 | 1.11 | 1.08 | 1.13 | 1.09 | 1.07 | 1.11*** |
| Richer | 4.44 | 1.15 | 1.13 | 1.18 | 1.14 | 1.12 | 1.17*** |
| Richest | 5.11 | 1.27 | 1.23 | 1.30 | 1.27 | 1.23 | 1.30*** |
| Residence |  | | | | | | |
| Urban | 4.70 | Reference |  |  |  |  |  |
| Rural | 3.68 | 1.003 | 0.985 | 1.021 | 1.018 | 0.999 | 1.036 |
| Model selection and adequacy | LR | -47377 | | | -46353 | | |
|  | AIC | 94818 | | | 92777 | | |
|  | BIC | 95078 | | | 93061 | | |

Key ANC= Antenatal care, NBR= negative binomial regression, ZINBR= Zero-inflated negative binomial regression # = number, ** =significant at p-value less than 0.01, *** =significant at p-value less than 0.001, AIRR= Adjusted incidence rate ratio LR = log likelihood Ratio, AIC =Akakian information Criteria, BIC= Bayesian information Criteria
