# Supplementary material for: Predictors and number of antenatal care visits among reproductive age women in Sub-Saharan Africa further analysis of recent demographic and health survey from 2017–2023: Zero-inflated negative binomial regression
Source: PLoS One. 2024 Oct 22;19(10):e0302297. doi: 10.1371/journal.pone.0302297 (PMC11495606; doi:10.1371/journal.pone.0302297)
Supplement: Attachment — Submitted filename: Response to Edittors and Reviewers.docx [file pone.0302297.s003.docx]

**Authors’ Responses to Editor and Reviewers Comments**

**Dear Academic Editor, Plose One**

“**Predictors and Number of Antenatal Care Visit among Reproductive Age Women in Sub-Saharan Africa Further Analysis of Recent** **DHS from 2017 -2023: Zero-Inflated Negative Binomial Regression**”

Dear Plos One Editors and Reviewers;

We are thankful for your constructive comments. We have looked at the comments and have revised our paper accordingly. We hope our paper improved as a result of incorporating reviewer’s and academic editor’s comments and suggestions. Here are authors’ responses to the comments.

Please find for your kind consideration the following:

1. A revised manuscript with track changes.
2. A revised paper without tracked changes
3. A rebuttal letter that responds to each point raised by the academic editor and reviewer.

The point by point responses of authors are written by hoping these changes would meet with your favorable consideration, we are happy to hear if there are more comments and suggestions. Please do not hesitate to let us know if you have any questions.

Your Sincerely

Mr. Abel Endawkie Correspondence Author

Department of Epidemiology and Biostatistics school of Public Health College of Medicine and Health science Wollo University Dessie Ethiopia

Tel. 251935459310	Email address abelendawkie@gmail.com

We have tried our best to improve it accordingly:

Please revise the manuscript.

Journal requirements:

When submitting your revision, we need you to address these additional requirements.

1. Please ensure that your manuscript meets PLOS ONE's style requirements, including those for file naming. The PLOS ONE style templates can be found at

<https://journals.plos.org/plosone/s/file?id=wjVg/PLOSOne_formatting_sample_main_body.pdf> and

<https://journals.plos.org/plosone/s/file?id=ba62/PLOSOne_formatting_sample_title_authors_affiliations.pdf>

**Response1:** We revised our manuscript based on Plos one submission guideline (manuscript, figure format and table format).

2. Your ethics statement should only appear in the Methods section of your manuscript. If your ethics statement is written in any section besides the Methods, please delete it from any other section.

**Response2:** we have prepared it.

**Point by point response of Authors for editors and reviewers comment**
**Editors comments to the Authors**

1. Please have a native English speaker review this manuscript for sentence and grammatical corrections. Also, carefully revise the manuscript following reviewers comments.

**Response1:** we are grateful to thank you for your Kindness and response. We tried to correct the language thoroughly.

**Reviewer Comment to the Authors**

**Reviewer#1	Comments**

Thanks for the opportunity to review this manuscript with the title “Predictors and number of Antenatal Care Visit among Reproductive Age Women in Sub-Saharan Africa Further Analysis of Recent DHS from 2017 -2023: Zero-Inflated Negative Binomial Regression” Where the aim was to determine mean number of ANC visit, proportion of at least eight ANC and its predictors among reproductive age women in Sub Saharan Africa using recent DHS data from 2017-2023 after the new WHO recommendation of ANC visit for a positive pregnancy experience.

**Response:** we are grateful to thank you for your Kindness and response. We had tried to correct your concern and suggestion thoroughly.

**General comments:**

**Comment1:** Mind a space before the references.

**Response1:** We would like to thank you kindly for your deep comment. We had revised and corrected it.

**Comment2:** Try to use a clearer language so that sentences are not miss-understood. Clear out misspelling. For example “visits were more for women who have primary” It should be had instead of have. And higher frequency instead of “education had more frequency”. Read the whole text and correct the language.

**Response2:** We would like to thank you kindly for your deep comment. We had revised and corrected it. See line number 222

**Response for general comment:** We would like to thank you kindly for your deep comment. We had revised and corrected it.

**Comment for** Abstract

**Comment3:** AIRR is written without an explanation.

**Response 3:** We would like to thank you kindly for your deep comment. We had revised and corrected it. See line number 31

**Comment4:** “were statistically significant with the numbers of ANC visits among reproductive age women in Sub-Saharan.” – write “associated with” instead of “were statistically significant with”.

**Response 4:** We would like to thank you kindly for your deep comment. We had revised and corrected it. See line number 37

**Comment for** Introduction

**Comment5:** This sentence is hard to understand. “It is a sequence of clinical tests and interventions that intend to make certain the well-being of each mother and the fetus at some point of being pregnant (2, 3).” Please re-write.

**Response5:** We would like to thank you for your deep comment and we would like to say sorry for confusing you in writing. We had revised and corrected it. See line number 52-55

**Comment for** Methods

**Comment6**. Wo

**Response6:** We really appreciated any concern you raise. However, we cannot understand the word (Wo)? We are open to receive more elaboration of your concern if it is serious and do not hesitate to communicate.

**Comment7.** In table 3 there are adjusted analyzes, however I can’t find in the text which variables they are adjusted for. Explain, and in addition explain your variables too.

**Response7:** We would like to thank you for your deep comment. We had written and included in the method of analysis. The adjusted analysis was conducted after Bivariable negative binomial regression analysis was conducted. Variables which had p-value<0.25 at Bivariable negative binomial regression analysis were selected for both multivariable standard and zero-inflated negative binomial regression.

**Comment8.** How come you don’t need an ethical permit? It is written that the women were interviewed or do I misunderstand?

**Response8:** We would like to thank you for your deep comment. No ethical approval was needed because we had used the demographic and health survey which identifies all data before making it public, and the used DHS data sets are openly accessible at https://dhsprogram.com/.

**Comment:** Results

**Comment9.** Table 1 is hard to read. Std error doesn´t have to be included. Frequency and percentage can be one column as well as the upper and lower value of the confidence interval.

**Response9:** We would like to thank you for your deep comment and we would like to apologize for we confused you in writing. We had revised and corrected it. See Table 1

**Comment10.** Try to condense the result, the most important numbers can be explained in the text, the rest is said by the table.

**Response10:** We would like to thank you for your deep comment. We had revised and corrected it.

**Comment11.** Row 227-230, comment the table, do not repeat it.

**Response11:** We would like to thank you for your deep comment. We had revised and corrected it.

**Comment12.** “Out of 188,880 women who were pregnant before the survey, 13579 (7.28%) had eight or more ANC visits between 2017 and 2023 in Sub-Saharan Africa (Table 2).” Do you mean ANC visits per pregnancy during the time of ANC during the whole time?

**Response12:** We would like to thank for your deep comment and we would like to apologize for having confused you in writing. This is for interviewed reproductive-age women who were pregnant and gave birth five years before the survey (in general it is not per pregnancy rather for the last one pregnancy before the survey).

**Comment for** Discussion

**Comment13.** “predictors of numbers of ANC visits” – How did you test which variables are predictors? Do you test the magnitude of the predictors?

**Response13:** We would like to thank you for your deep comment. First for more clear understanding in this study predictors were considered as factors/determinant for numbers of ANC visit among reproductive-age women. The reason why we are using the term predictor is more appropriate for count model analysis than using associated factors or determinants or covariates.

We had not conducted predictive model analysis. We had conducted a count model analysis to determine the mean number of ANC visits and factors for low number of ANC visits and higher number of ANC visits among reproductive women who were pregnant and gave birth before the survey. We had measured the magnitude of each factor/predictors contributed for ANC visit using count by incidence rate ratio. As you know incidence rate ratio (IRR) is a measure of association or effect measure which is used to measure the relationship between outcome and independent variable.

**Comment514.** Please re right: “One explanation would be that those women who have educated husbands will also likely to understand themselves and possibly this may be due to support by their partner (husband)”.

**Response14:** We would like to thank you for your deep comment and we would like to apologize for we confused you in writing. We had revised and corrected it.

**Comment 15.** There are low number of ANC visits. Which are the consequences? Please tell us something about the number of ANC and fetal outcomes.

**Response15:** We would like to thank you for your deep comment. In this study we had conducted to determine the factors for low number of ANC visits and higher number of ANC visits among reproductive women who were pregnant and gave birth before the survey. We have not measured the consequence of the low number of ANC visits. However, in the study we had conducted entitled ‘’number of pregnancy loss and associated factors among reproductive age women who were ever pregnant before the survey in Sub-Saharan Africa using DHS data’’ showed that there is the high number of pregnancy loss among women who had low number of ANC visit and poor quality of ANC.

As you know in general, obviously a low number of ANC visits will result in poor fetal outcomes like abortion, still birth, low birth weight, neonatal mortality. This is stated in the introduction part as the benefit, impact/ consequence of ANC on maternal and newborn health.

**Comment16.** What could be done? What are your suggestions of action to increase the number of ANC?

**Response16:** We would like to thank you for your deep comment. The possible recommendation of to increase number of ANC visit based on our finding:

1. Efforts should be geared towards improving maternal and husband’s educational status.
2. We strongly recommend that the governments of Sub-Saharan Africa countries should empower women economically and educationally so as to achieve the goals of ANC as recommended by the WHO.

**Reviewer#2 Comment**

The identified spelling and grammatical errors have been highlighted appropriately in the relevant pages of the text for ease of reference and correction. Some sentences were unnecessarily long with repetitions and ambiguous. I have provided some corrections while leaving the ambiguous (unclear) ones for the Authors to resolve.
**Response:** We would like to thank you for your kind response and deep comment. We have revised and corrected it.

**Reviewer#3 Comment**

Good effort. However, I need English language editing like in line 249. State limitations of study. List the Sub-Saharan-African nations involved in the study. Authors may engage an English language editor.

**Response:** We would like to thank you for your kind response and deep comment. We have revised and corrected it. The study relies on secondary data from the Demographic and Health Surveys (DHS), which may have limitations in terms of data accuracy and reliability. The quality of the data depends on the collection methods and the respondents' ability to accurately recall and report information. The study relies on self-reported data, which may be subject to recall bias or social desirability bias. Participants may over-report or under-report their ANC visits due to social expectations or memory limitations.

The study design is cross-sectional, which limits the ability to establish causal relationships between the predictors and the number of ANC visits.

We had listed the countries included in this data analysis in the data source section of methods. See line number 102-103.

**Reviewer #4 comment:**

Thank you for providing such an insightful research article. I appreciate the depth of your work. I would like to offer some constructive feedback and inquiries.

**Comment1:** 1- Have you analyzed the correlation between the number of antenatal care (ANC) visits and gestational age?

**Response1:** We would like to thank you for your kind response and deep comment. Since we had analyzed secondary data from publically available. The data set has no gestational age related data because it was collected among reproductive-age women who were pregnant and gave birth five years before the survey. As a result, we had not analyzed the correlation between the number of antenatal care (ANC) visits and gestational age. Our objective was to determine the mean number of ANC visits, proportion of at least eight ANC visits and its predictors among reproductive age women in Sub Saharan Africa using recent DHS data from 2017-2023 following the new WHO recommendation of ANC visit for a positive pregnancy experience.

**Comment2-** Could you elaborate on the antenatal care settings within each country included in the study? Additionally, it would be beneficial to elucidate any specific systems implemented to ensure patient attendance at these visits. Furthermore, providing clarity on the healthcare professionals typically involved in conducting these visits—whether it be midwives, nurses, or obstetricians—would enhance the understanding of the study's feasibility.

**Response2-** We would like to thank you for your kind response and deep comment.

1. Yes we had tried to elaborate in the result and discussion section As you in table 1 of the result there is the disparity of the average number of ANC visits in the regions of SSA. The mean number of ANC visits was lowest in Central Africa and highest in West Africa. These disparities could be due to inequalities in the accessibility of maternal health services, poor or absent transportation, inequality in the number of healthcare providers, and disparities in access to education in the countries of these regions. Additionally, the differences in the implementation of maternal and child health programs, women’s education, and the role of women in household wealth status across different countries of SSA could also contribute to these disparities.
2. We believe that the difference in health care professionals involved in conducting the ANC visit have an impact on the quality of ANC service intern may have an impact on the number of ANC visits. In this study health care professionals involved in attending ANC visits during pregnancy like midwives, nurses, health officers, medical doctors, or obstetricians were not assessed due to incompleteness of data set may be a limitation of study.

**Comment3-** Have you measured the frequency of visits against the different medical conditions associated with pregnancy, and whether this pregnancy is low risk or high risk?

**Response3:** We would like to thank you for your kind response and deep comment. We really appreciate your concern and it is important to know it for maternal and child health.

Since our objective was to determine the mean number of ANC visits, the proportion of at least eight ANC and its predictors among reproductive age women in Sub Saharan Africa using recent DHS data from 2017-2023 following the new WHO recommendation of ANC visit for a positive pregnancy experience. We had not measured the frequency of visits against the different medical conditions associated with pregnancy, and whether this pregnancy is low risk or high risk? However, in the study we had conducted entitled ‘’number of pregnancy loss and associated factors among reproductive age women who were ever pregnant before the survey in Sub-Saharan Africa using DHS data’’ showed that there is a high number of pregnancy loss risk among women who had low number of ANC visits and poor quality of ANC.

As you know in general, obviously a low number of ANC visits will result in poor fetal outcomes like abortion, stillbirth, low birth weight, and neonatal mortality.

**We feel you may understand our interest.**

**Comment4-** Are there discernible reasons behind the less frequent ANC visits observed in your findings?.

**Response4:** We would like to thank you for your deep comment. Yes in this study as you read on the result and discussion, there are identified discernible reasons for low ANC visits.

1. Younger age of women: Younger women may face barriers such as lack of awareness, limited decision-making power, or societal norms that discourage seeking ANC may lead to less frequent ANC visits.
2. Unplanned pregnancy: Unplanned, may influence ANC utilization. Women with unplanned pregnancies may be less likely to seek regular ANC visits.
3. Birth order: Women with higher birth order may have competing priorities or responsibilities, which can affect their ability to attend ANC visits.
4. Household size: The size of the household was identified as a predictor of ANC visits. Women from larger households may face challenges in accessing healthcare services due to limited resources or logistical constraints.
5. Number of under-five children: The presence of other children under the age of five in the household may influence ANC utilization. Women with multiple young children may have increased caregiving responsibilities, making it more difficult for them to attend ANC visits.
6. Wealth index: The wealth index, which is a measure of socioeconomic status, was found to be associated with ANC visits. Women from lower socioeconomic backgrounds may face financial barriers or lack of access to transportation, which can hinder their ability to attend ANC visits

**Comments5-** Do you have any recommendations based on your study's findings?

**Response5**: We would like to thank you for your deep comment. The possible recommendation of to increase number of ANC visit based on our finding:

1.	Efforts should be geared towards improving maternal and husband’s educational status.

2.	We strongly recommend that the governments of Sub-Saharan Africa countries should empower women economically and educationally so as to achieve the goals of ANC as recommended by the WHO.

**Comment-** Including a section on the limitations of your study would be instrumental in providing a comprehensive understanding of its scope and potential constraints.

**Response6:** We are grateful to thank you for your Kindness and response. We revised it

**We feel all of you may understand our feeling and interest.**

**We are thankful for all really**
