# Supplementary material for: Predictors and number of antenatal care visits among reproductive age women in Sub-Saharan Africa further analysis of recent demographic and health survey from 2017–2023: Zero-inflated negative binomial regression
Source: PLoS One. 2024 Oct 22;19(10):e0302297. doi: 10.1371/journal.pone.0302297 (PMC11495606; doi:10.1371/journal.pone.0302297)
Supplement: Attachment — Submitted filename: Authors response to edittors and reviewers (Abel).docx [file pone.0302297.s004.docx]

**Authors’ Responses to Editor and Reviewers Comments**

**Dear Academic Editor, Plose One**

“**Predictors and Number of Antenatal Care Visit among Reproductive Age Women in Sub-Saharan Africa Further Analysis of Recent** **DHS from 2017 -2023: Zero-Inflated Negative Binomial Regression**”

Dear Plos One Editors and Reviewers;

We are thankful for your constructive comments. We have looked at the comments and have revised our paper accordingly. We hope our paper improved as a result of incorporating the reviewer’s and academic editor’s comments and suggestions. Here are the authors’ responses to the comments.

Please find for your kind consideration the following:

1. A revised manuscript with track changes.
2. A revised paper without tracked changes
3. A rebuttal letter that responds to each point raised by the academic editor and reviewer.

The point by point responses of authors are written by hoping these changes would meet with your favorable consideration, we are happy to hear if there are more comments and suggestions. Please do not hesitate to let us know if you have any questions.

Yours Sincerely

Mr. Abel Endawkie Correspondence Author

Department of Epidemiology and Biostatistics School of Public Health College of Medicine and Health Science Wollo University Dessie Ethiopia

Tel. 251935459310	Email address abelendawkie@gmail.com

We have tried our best to improve it accordingly:

Please revise the manuscript.

Journal requirements:

**Point-by-point response of Authors for editor's and reviewers' comment**
**Editor's comments to the Authors**

**Comment:** Please respond to all reviewers' comments including those from the previous review which you ignored. Also, submit a rebuttal letter containing the responses to the reviewers' comments and highlight this in the main manuscript. I strongly suggest you have a native English speaker review this manuscript for grammatical errors.

**Response:** We are grateful to thank you for your Kindness and response. We tried to correct the language thoroughly.

**Requesting of understanding**

First and foremost, we apologize for any inconvenience caused during the submission of the revised manuscript. Unfortunately, we inadvertently mixed the last revised version with the initial version. During the submission of the first revision, we failed to remove the initial version of the Manuscript in the online submission of our revision, which led to the concern raised by the second reviewer (reviewer #2). Despite our sincere efforts to address reviewer#2 feedback, our response to his comment was not reached. We kindly request to resubmit the manuscript with track changes reflecting the first revision in our current revision.

**Authors Response to the Reviewer Comment**

**Reviewer #1:** Thanks for your answers, corrections, and changes. I'm satisfied with the answers and explanations.

**Response 1:** We sincerely appreciate your kindness and contribution to improving our manuscript. All the ideas you’ve raised are valuable for enhancing our work in the scientific context.

**Reviewer #2:** Although most of the grammatical/spelling/typographical errors have been substantially addressed, some were either deliberately IGNORED or not NOTICED. Their correction would

**Response:** We sincerely apologize that the corrected version of our document did not reach you. Dear Reviewer, we deeply appreciate your insightful comments, which are invaluable for ensuring the scientific validity of our manuscript. We sincerely thank you for your patience and apologize for any inconvenience caused by the submission of our revised document. During the revision submission process, where the revised version became intermixed with our initial document. Please rest assured that we made every effort to address your feedback and suggestions during the revision. Specifically, we incorporated your recommended changes to words and sentences, including the correction you pointed out in the review of our manuscript: For example “The research was conducted a statistical analysis using…” which we promptly adjusted to “The research was conducted as a statistical analysis using…” at the beginning of our discussion. Thank you once again for your valuable input.

**Comment 1:** For example, in the discussion section, line 249 reads: "The research was conducted a statistical analysis using ......" I corrected this to read "The research was conducted AS a statistical analysis using.... but this correction was ignored.

**Response 1:** We deeply appreciate your insightful comments. In response to your suggestion, we deemed it important to make the sentence change. Accordingly, we revised and corrected it as it is. See line # 31

**Comment2:** Similarly, lines 288-291 starting with "To promote frequent antenatal care ........ " is ambiguous, especially with the word "INTERN" and i suggested its revision(with a guide provided for this) but this was also IGNORED.

**Response 2:** We deeply appreciate your insightful comments. In response to your suggestion, we deemed it important to make the sentence change. Accordingly, we revised and corrected it as it is. See line # 325

**Comment 3:** Lines 325-331 were similarly AMBIGUOS and a suggestion made for its revision was IGNORED. It does not make intelligent reading as presently written and does not convey much.

**Response 3:** We deeply appreciate your insightful comments. In response to your suggestion, we deemed it important to make the sentence change. Accordingly, we revised and corrected it as it is. See line # 326-328

**Comment4:** Line 128 under sampling method, the word "COMPLIED" was corrected to read "COMPILED" but this was IGNORED: See fig1

**Response 4:** We deeply appreciate your insightful comments. In response to your suggestion, we deemed it important to make the sentence change. Accordingly, we revised and corrected as it is. As a reminder, we have made the necessary corrections. The figure has been submitted separately as **“Fig1”** in **“TIF”** format, and you can find it. See fig1

**Comment5:** In Table 1 under "WANTED (PREGNANCY TYPE) the word WNWANTED was corrected to UNWANTED but this was ignored!

**Response 5:** We deeply appreciate your insightful comments. In response to your suggestion, we deemed it important to make the sentence change. Accordingly, we revised and corrected it as it is. You can find it in our revised version of the table in the manuscript. See table 1 and 2

**Comment6:** A suggestion for abbreviations to be written in full at first mention was also IGNORED. There are a few others which i chose not to point out here but which were highlighted in RED in my previous review and which are intended to make the English intelligible to read.

**Response 6:** We deeply appreciate your insightful comments. In response to your suggestion, we deemed it important to make the sentence change. Accordingly, we revised and corrected it as it is. You can find it in our revised version of the manuscript.

**Reviewer #3:** I am not aware that the authors have stated whether all 40 countries in Sub-Saharan Africa wee involved in the assessment. Authors should mention the countries within the subregion.

**Responses**: We greatly appreciate your insightful comments. In response to your feedback, we initially attempted to include the specific country in our analysis during our first revision. In the second round revised version of our manuscript, we have included the countries within the sub-region (four regions of Africa) in the data source section of the method.

**Reviewer #4:** I couldn't find any evidence that the previous reviewer comments have been addressed. I suggest attaching a file containing the responses to the reviewers' comments and highlighting this on the main manuscript as well.

**Response:** We greatly appreciate your input. Honestly, we attached our response to all reviewers’ comments, which was saved as a file titled “Author Response to Reviewers’ Comments during the initial Revision. Now, we have included the author’s response to the reviewer’s comments in the revised manuscript below for your information.

**Authors Response to the Reviewer Comment in the First Revision of Manuscript**

**Reviewer#1	Comments**

Thanks for the opportunity to review this manuscript with the title “Predictors and number of Antenatal Care Visit among Reproductive Age Women in Sub-Saharan Africa Further Analysis of Recent DHS from 2017 -2023: Zero-Inflated Negative Binomial Regression” Where the aim was to determine mean number of ANC visit, proportion of at least eight ANC and its predictors among reproductive age women in Sub Saharan Africa using recent DHS data from 2017-2023 after the new WHO recommendation of ANC visit for a positive pregnancy experience.

**Response:** we are grateful to thank you for your Kindness and response. We have tried to correct your concern and suggestion thoroughly.

**General comments:**

**Comment1:** Mind a space before the references.

**Response 1:** We would like to thank you kindly for your deep comment. We had revised and corrected it.

**Comment2:** Try to use a clearer language so that sentences are not miss-understood. Clear out misspelling. For example “visits were more for women who have primary” It should be had instead of have. And higher frequency instead of “education had more frequency”. Read the whole text and correct the language.

**Response 2:** We would like to thank you kindly for your deep comment. We have revised and corrected it. See line number 222

**Response for general comment:** We would like to thank you kindly for your deep comment. We have revised and corrected it.

**Comment for** Abstract

**Comment3:** AIRR is written without an explanation.

**Response 3:** We would like to thank you kindly for your deep comment. We have revised and corrected it. See line number 31

**Comment4:** “were statistically significant with the numbers of ANC visits among reproductive age women in Sub-Saharan.” – write “associated with” instead of “were statistically significant with”.

**Response 4:** We would like to thank you kindly for your deep comment. We have revised and corrected it. See line number 37

**Comment for** Introduction

**Comment5:** This sentence is hard to understand. “It is a sequence of clinical tests and interventions that intend to make certain the well-being of each mother and the fetus at some point of being pregnant (2, 3).” Please re-write.

**Response 5:** We would like to thank you for your deep comment and we would like to say sorry for confusing you in writing. We have revised and corrected it. See line number 52-55

**Comment for** Methods

**Comment6**. Wo

**Response 6:** We really appreciate any concern you raise. However, we cannot understand the word (Wo)? We are open to receiving more elaboration of your concern if it is serious and do not hesitate to communicate.

**Comment7.** In table 3 there are adjusted analyses, however, I can’t find in the text which variables they are adjusted for. Explain, and in addition explain your variables too.

**Response 7:** We would like to thank you for your deep comment. We had written and included in the method of analysis. The adjusted analysis was conducted after Bivariable negative binomial regression analysis was conducted. Variables that had p-value<0.25 at Bivariable negative binomial regression analysis were selected for both multivariable standard and zero-inflated negative binomial regression.

**Comment8.** How come you don’t need an ethical permit? It is written that the women were interviewed or do I misunderstand?

**Response8:** We would like to thank you for your deep comment. No ethical approval was needed because we had used the demographic and health survey which identifies all data before making it public, and the used DHS data sets are openly accessible at https://dhsprogram.com/.

**Comment:** Results

**Comment9.** Table 1 is hard to read. Std error doesn´t have to be included. Frequency and percentage can be one column as well as the upper and lower value of the confidence interval.

**Response9:** We would like to thank you for your deep comment and we would like to apologize for confusing you in writing. We have revised and corrected it. See Table 1

**Comment10.** Try to condense the result, the most important numbers can be explained in the text, and the rest is said by the table.

**Response10:** We would like to thank you for your deep comment. We have revised and corrected it.

**Comment11.** Row 227-230, comment the table, do not repeat it.

**Response11:** We would like to thank you for your deep comment. We have revised and corrected it.

**Comment12.** “Out of 188,880 women who were pregnant before the survey, 13579 (7.28%) had eight or more ANC visits between 2017 and 2023 in Sub-Saharan Africa (Table 2).” Do you mean ANC visits per pregnancy during the time of ANC during the whole time?

**Response 12:** We would like to thank you for your deep comment and we would like to apologize for having confused you in writing. This is for interviewed reproductive-age women who were pregnant and gave birth five years before the survey (in general it is not per pregnancy but rather for the last pregnancy before the survey).

**Comment for** Discussion

**Comment13.** “predictors of numbers of ANC visits” – How did you test which variables are predictors? Do you test the magnitude of the predictors?

**Response 13:** We would like to thank you for your deep comment. First for more clearer understanding of this study predictors were considered as factors/determinants for the numbers of ANC visits among reproductive-age women. The reason why we are using the term predictor is more appropriate for count model analysis than using associated factors determinants or covariates.

We had not conducted a predictive model analysis. We conducted a count model analysis to determine the mean number of ANC visits and factors for low number of ANC visits and higher number of ANC visits among reproductive women who were pregnant and gave birth before the survey. We measured the magnitude of each factor/predictor that contributed to ANC visit using count by incidence rate ratio. As you know incidence rate ratio (IRR) is a measure of association or effect measure which is used to measure the relationship between outcome and independent variable.

**Comment514.** Please re right: “One explanation would be that those women who have educated husbands will also likely to understand themselves and possibly this may be due to support by their partner (husband)”.

**Response14:** We would like to thank you for your deep comment and we would like to apologize for confusing you in writing. We have revised and corrected it.

**Comment 15.** There are low number of ANC visits. Which are the consequences? Please tell us something about the number of ANC and fetal outcomes.

**Response 15:** We would like to thank you for your deep comment. In this study, we determine the factors for low number of ANC visits and higher number of ANC visits among reproductive women who were pregnant and gave birth before the survey. We have not measured the consequence of the low number of ANC visits. However, the study we had conducted entitled ‘’Number of pregnancy loss and associated factors among reproductive age women who were ever pregnant before the survey in Sub-Saharan Africa using DHS data’’ showed that there is a high number of pregnancy loss among women who had the low number of ANC visit and poor quality of ANC.

As you know in general, obviously a low number of ANC visits will result in poor fetal outcomes like abortion, stillbirth, low birth weight, and neonatal mortality. This is stated in the introduction part as the benefit, impact/ consequence of ANC on maternal and newborn health.

**Comment16.** What could be done? What are your suggestions for action to increase the number of ANC?

**Response 16:** We would like to thank you for your deep comment. The possible recommendation to increase the number of ANC visits based on our findings:

1. Efforts should be geared towards improving maternal and husband’s educational status.
2. We strongly recommend that the governments of Sub-Saharan African countries should empower women economically and educationally to achieve the goals of ANC as recommended by the WHO.

**Reviewer#2 Comment**

The identified spelling and grammatical errors have been highlighted appropriately in the relevant pages of the text for ease of reference and correction. Some sentences were unnecessarily long with repetitions and ambiguous. I have provided some corrections while leaving the ambiguous (unclear) ones for the Authors to resolve.
**Response:** We would like to thank you for your kind response and deep comment. We have revised and corrected it.

**Reviewer#3 Comment**

Good effort. However, I need English language editing like in line 249. State limitations of study. List the Sub-Saharan-African nations involved in the study. Authors may engage an English language editor.

**Response:** We would like to thank you for your kind response and deep comment. We have revised and corrected it. The study relies on secondary data from the Demographic and Health Surveys (DHS), which may have limitations in terms of data accuracy and reliability. The quality of the data depends on the collection methods and the respondents' ability to accurately recall and report information. The study relies on self-reported data, which may be subject to recall bias or social desirability bias. Participants may over-report or under-report their ANC visits due to social expectations or memory limitations.

The study design is cross-sectional, which limits the ability to establish causal relationships between the predictors and the number of ANC visits.

We had listed the countries included in this data analysis in the data source section of methods. See line number 102-103.

**Reviewer #4 comment:**

Thank you for providing such an insightful research article. I appreciate the depth of your work. I would like to offer some constructive feedback and inquiries.

**Comment1:** 1- Have you analyzed the correlation between the number of antenatal care (ANC) visits and gestational age?

**Response 1:** We would like to thank you for your kind response and deep comment. Since we had analyzed secondary data from publically available, the data set has no gestational age-related data because it was collected among reproductive-age women who were pregnant and gave birth five years before the survey. As a result, we did not analyze the correlation between the number of antenatal care (ANC) visits and gestational age. Our objective was to determine the mean number of ANC visits, the proportion of at least eight ANC visits, and its predictors among reproductive-age women in Sub-Saharan Africa using recent DHS data from 2017-2023 following the new WHO recommendation of ANC visits for a positive pregnancy experience.

**Comment2-** Could you elaborate on the antenatal care settings within each country included in the study? Additionally, it would be beneficial to elucidate any specific systems implemented to ensure patient attendance at these visits. Furthermore, providing clarity on the healthcare professionals typically involved in conducting these visits—whether it be midwives, nurses, or obstetricians—would enhance the understanding of the study's feasibility.

**Response 2-** We would like to thank you for your kind response and deep comment.

1. Yes, we had tried to elaborate in the result and discussion section As you see in Table 1 of the result there is the disparity of the average number of ANC visits in the regions of SSA. The mean number of ANC visits was lowest in Central Africa and highest in West Africa. These disparities could be due to inequalities in the accessibility of maternal health services, poor or absent transportation, inequality in the number of healthcare providers, and disparities in access to education in the countries of these regions. Additionally, the differences in the implementation of maternal and child health programs, women’s education, and the role of women in household wealth status across different countries of SSA could also contribute to these disparities.
2. We believe that the difference in health care professionals involved in conducting the ANC visit has an impact on the quality of ANC service interns and may have an impact on the number of ANC visits. In this study health care professionals involved in attending ANC visits during pregnancy like midwives, nurses, health officers, medical doctors, or obstetricians were not assessed due to the incompleteness of the data set may be a limitation of the study.

**Comment3-** Have you measured the frequency of visits against the different medical conditions associated with pregnancy, and whether this pregnancy is low risk or high risk?

**Response 3:** We would like to thank you for your kind response and deep comment. We appreciate your concern and it is important to know it for maternal and child health.

Our objective was to determine the mean number of ANC visits, the proportion of at least eight ANC, and its predictors among reproductive-age women in Sub-Saharan Africa using recent DHS data from 2017-2023 following the new WHO recommendation of ANC visits for a positive pregnancy experience. We had not measured the frequency of visits against the different medical conditions associated with pregnancy, and whether this pregnancy is low risk or high risk. However, the study we had conducted entitled ‘’Number of pregnancy loss and associated factors among reproductive age women who were ever pregnant before the survey in Sub-Saharan Africa using DHS data’’ showed that there is a high number of pregnancy loss risk among women who had low number of ANC visits and poor quality of ANC.

As you know in general, obviously a low number of ANC visits will result in poor fetal outcomes like abortion, stillbirth, low birth weight, and neonatal mortality.

**We feel you may understand our interest.**

**Comment4-** Are there discernible reasons behind the less frequent ANC visits observed in your findings?.

**Response 4:** We would like to thank you for your deep comment. Yes in this study as you read on the result and discussion, there are identified discernible reasons for low ANC visits.

1. Younger age of women: Younger women may face barriers such as lack of awareness, limited decision-making power, or societal norms that discourage seeking ANC may lead to less frequent ANC visits.
2. Unplanned pregnancy: Unplanned, may influence ANC utilization. Women with unplanned pregnancies may be less likely to seek regular ANC visits.
3. Birth order: Women with higher birth order may have competing priorities or responsibilities, which can affect their ability to attend ANC visits.
4. Household size: The size of the household was identified as a predictor of ANC visits. Women from larger households may face challenges in accessing healthcare services due to limited resources or logistical constraints.
5. Number of under-five children: The presence of other children under the age of five in the household may influence ANC utilization. Women with multiple young children may have increased caregiving responsibilities, making it more difficult for them to attend ANC visits.
6. Wealth index: The wealth index, which is a measure of socioeconomic status, was found to be associated with ANC visits. Women from lower socioeconomic backgrounds may face financial barriers or lack of access to transportation, which can hinder their ability to attend ANC visits

**Comments5-** Do you have any recommendations based on your study's findings?

**Response 5**: We would like to thank you for your deep comment. The possible recommendation to increase the number of ANC visits based on our findings:

1.	Efforts should be geared towards improving maternal and husband’s educational status.

2.	We strongly recommend that the governments of Sub-Saharan African countries should empower women economically and educationally to achieve the goals of ANC as recommended by the WHO.

**Comment-** Including a section on the limitations of your study would be instrumental in providing a comprehensive understanding of its scope and potential constraints.

**Response6:** We are grateful to thank you for your Kindness and response. We revised it

**We feel all of you may understand our feelings and interests.**

**We are thankful for all!!**
