# Supplementary material for: Predictors and number of antenatal care visits among reproductive age women in Sub-Saharan Africa further analysis of recent demographic and health survey from 2017–2023: Zero-inflated negative binomial regression
Source: PLoS One. 2024 Oct 22;19(10):e0302297. doi: 10.1371/journal.pone.0302297 (PMC11495606; doi:10.1371/journal.pone.0302297)
Supplement: Attachment — Submitted filename: Authors response to edittors and reviewers (Abel).docx [file pone.0302297.s005.docx]

**Authors’ Responses to Editor and Reviewers Comments**

**Dear Academic Editor of Plose One**

“**Predictors and Number of Antenatal Care Visits among Reproductive Age Women in Sub-Saharan Africa Further Analysis of Recent** **DHS from 2017 -2023: Zero-Inflated Negative Binomial Regression**”

Dear Plos One Editors and Reviewers;

We are thankful for your constructive comments. We have looked at the comments and have revised our paper accordingly. We hope our paper improved as a result of incorporating the reviewer’s and academic editor’s comments and suggestions. Here are the authors’ responses to the comments.

Please find for your kind consideration the following:

1. A revised manuscript without track changes.
2. A revised paper with tracked changes
3. A rebuttal letter that responds to each point raised by the academic editor and reviewer.

The point-by-point responses of authors are written by hoping these changes would meet with your favorable consideration, we are happy to hear if there are more comments and suggestions. Please do not hesitate to let us know if you have any questions.

Yours Sincerely

Mr. Abel Endawkie Correspondence Author

Department of Epidemiology and Biostatistics School of Public Health College of Medicine and Health Science Wollo University Dessie Ethiopia

Tel. 251935459310	Email address abelendawkie@gmail.com

We have tried our best to improve it accordingly:

Please revise the manuscript.

Journal requirements:

**Point-by-point response of Authors for editor's and reviewers' comment**
**Editor's comments to the Authors**

Additional Editor Comments:

During final checks of this article, concerns were raised that the statistical analyses may not have been evaluated in sufficient detail during peer review. To ensure that your work receives a thorough and rigorous review, the manuscript has now been assessed by one of our statistical advisors. Please accept our apologies that this review was not arranged at an earlier stage in the review process.

**Response:** We sincerely appreciate your kindness, understanding, and prompt response. We kindly accept your apologies for any inconvenience caused by the delayed review process. Rest assured, our manuscript is now receiving the thorough evaluation it deserves. If you have any further questions or concerns, please don’t hesitate to reach out.

**In addition to the reviewer comments, please note the following concerns:**

**Comment 1.** The manuscript does not explicitly reference the DHS instrument(s) included in the analysis. Details of the specific countries and years should be included

**Response 1:** We are grateful to thank you for your kindness and response. We have tried to correct your concern and suggestion thoroughly. We have attached the details of the specific countries and years as supplementary files.

**Comment 2**. The manuscript does not adequately cite and discuss in the Introduction any previous literature pooling DHS surveys to investigate predictors and number of antenatal care visits in sub-Saharan Africa. The manuscript should cite and discuss existing literature, and indicate how this study will build on the existing literature, in line with our 2nd publication criterion.

**Response 2:** We sincerely appreciate your kindness and prompt response. We have diligently addressed your concerns and suggestions

**Authors Response to the Reviewer Comment**

**Review Comments to the Author**

**Reviewer #1:** I have got resonably answers to my comments and these were adequate and enhanced the project further.

**Response:** We sincerely appreciate your kindness and prompt response. Your valuable insights, comments, and suggestions for improving the scientific plausibility of our manuscript are highly appreciated.

**Reviewer #2:** The Authors have further addressed the issues i raised in my earlier review and effected the grammatical and typographical errors earlier highlighted. I recommend the acceptance of the manuscript for publication.

**Response:** We sincerely appreciate your kindness and prompt response. Your valuable insights, comments, and suggestions for improving the scientific plausibility of our manuscript are highly appreciated.

**Reviewer #4:** Thank you for your contribution to the current research field and your dedication towards addressing the previous reviewers' comments.

**Response:** We sincerely appreciate your kindness and prompt response. Your valuable insights, comments, and suggestions for improving the scientific plausibility of our manuscript are highly appreciated.

**Reviewer #5:** The authors studied variables associated with mean number of antenatal care among productive women Sub-Saharan Africa using data collected from 2017-2023 Demographic and Health Surveys (DHS).

**I have some questions about the statistical analysis.**

**Comment 1:** Did all pregnancies of the same women have separate entries in the database?

**Response 1:** We sincerely appreciate your insightful comment and questions and we apologize for any confusion in our writing. Our study focuses on reproductive-age women who were pregnant and gave birth to determine the mean number of ANC vistas and its predictors during the last one/recent one pregnancy before the survey. Our study is not to each and all pregnancies before the survey but rather certainly we focused on the last one pregnancy before the survey.

**Comment 2:** Due to the clustering of the data at several levels- pregnant women, household, sampling cluster, a multilevel mixed effects model should be used to account for the within clustering correlation.

**Response 2:** We sincerely appreciate your insightful comment, and we apologize for any confusion in our writing. Before we conducted this analysis we had checked the cluster variation since the DHS data is hierarchical which assumes the pregnant women and their households are nested within the enumeration area (clusters). This suggests that pregnant women in households with similar characteristics can have different health outcomes when residing in different communities(clusters) with different characteristics. Therefore, to check whether there is distinct measures of variation and any potential clustering effect for conducting multilevel analysis: the intra-class correlation coefficient (ICC) was calculated, and ICC=3.2% which is less than 10% and even 5% which favors the individual level analysis using negative binomial regression analysis.

**Comment 3:** More details are needed for the intra-class correlation coefficient calculation in lines 171-173. Please define sigma squared and pi squared. How were the values of sigma squared and pi squared obtained, estimates from a mixed model?

**Response 3:** We sincerely appreciate your insightful comment, and we apologize for any confusion in our writing. **Here is the explanation.** The intra-class correlation coefficient (ICC) was calculated as ICC = $\frac{\delta2}{\delta2+ \pi2/ 3}$ where δ2 indicates the estimated constant variance of clusters in the null model( obtained from variance components of the null model) and π2/ 3= 3.29 is the variance of the model with scale parameter.

**Comment 4:** What variables were used as predictors for the zero component of the zero-inflated negative binomial model? Were any of the predictors significant?

**Response 4:** We sincerely appreciate your deep, insightful, valuable comments and questions, and we apologize for any confusion in our writing. In the context of a zero-inflated negative binomial (ZINB) model, the selection of predictors for the zero component involves identifying factors that influence the probability of observing a zero count (excess zeros). The zero component of a ZINB model captures the excess zeros in the data. These zeros may arise due to a separate process (e.g., structural zeros) rather than the underlying count distribution.

As you know the selection of a predictor for the zero component of the ZINB model is based on considering potential predictors that might influence the excess zeros.

In general, there are common predictors including Covariates like socio-demographic, individual-level, or environmental variables related to the study population. Contextual factors like region (place of residence), time, or other contextual information and Study-Specific Variables like Treatment group, and intervention.

In our study, since it is secondary data collected using a cross-sectional study approach which has no follow-up time or specified intervention in the data.

The selected predictors of zero component in our analysis were socio-demographic, individual-level factors, place of residence for zero component to account the excess zeros in the data.

**Yes there are significant variable for zero ANC visit.** From selected factors of zero component of zero-inflated negative binomial (ZINB) model, maternal educational and occupational status, husbands occupational status, unwanted pregnancy, no more fertility desire, media exposure, wealth index, and rural residences significantly associated with zero ANC visits either negatively or positively.

Here is the command used for Zero-inflated negative binomial regression in Stata 17. “zinb m14(number of ANC) i.v013(maternal age ) i.v106(maternal educational status) i.respoworkings(maternal occupational status) i.Haseduca(husband educational status) i.hasbanworkstatus_cat(husband educational status) i.wantedpreg(pregnancy type) i.fertilityprefernc_cat (fertility preference) i.mediaexposure(media exposure) birth order ( birth order) v136(household number) v137 (numbers of under-five child) i.v151(sex of household head) i.HHHage(age of household head) i.v190(wealth index) i.v025 (place of residence) [iweight =wt],**inflate(** i.v106(maternal educational status) i.respoworkings(maternal occupational status) i.Haseduca(husband educational status) i.hasbanworkstatus_cat(husband educational staus) i.wantedpreg(pregnancy type) i.fertilityprefernc_cat (fertility preference) i.mediaexposure(media exposure) birth order ( birth order) v136(household number) v137 (numbers of under-five child) i.v151(sex of household head) i.HHHage(age of household head) i.v190(wealth index) i.v025 (place of residence) irr iterate(300) nolog”

**Comment 5:** Figure 1. What do the two numbers 191,344 and 188,880 stand for respectively?

**Response 5:** We sincerely appreciate your insightful, and valuable questions. The 191,344 women mean the unweighted total sample size of screened (eligible) reproductive women obtained from the included dataset. The 188,880 indicates the weighted sample size of this study obtained by dividing those eligible reproductive women (V005) by 1,000,000 (V005/1000000) to account for unequal probability of sampling to reduce sample variability by the principle of the DHS program using sample weights before further analysis.

**Comment 6:** Does “5 years before the survey” means “in the 5 years preceding the survey”?

**Response 6:** We are grateful to thank you for your kindness and response. We sincerely appreciate your deep, insightful, and valuable comment, and we apologize for any confusion in our writing. This study includes data collected in the DHS among reproductive-age women who were pregnant and gave birth in the last 5 years before the survey.

**We feel all of you may understand our feelings and interests.**

**We are thankful for all!!**
